# IoT Security Configurability with Security-by-Contract

**DOI:** 10.3390/s19194121

**Published:** 2019-09-23

**Authors:** Alberto Giaretta, Nicola Dragoni, Fabio Massacci

**Affiliations:** 1 Centre for Applied Autonomous Sensors Systems (AASS), Örebro University, 701 82 Örebro, Sweden; 2 DTU Compute, Technical University of Denmark, 2800 Kgs. Lyngby, Denmark; 3 Department of Information Science and Engineering, University of Trento, 38123 Trento, Italy

**Keywords:** IoT, configurability, Fog computing, security-by-contract, security

## Abstract

Cybersecurity is one of the biggest challenges in the Internet of Things (IoT) domain, as well as one of its most embarrassing failures. As a matter of fact, nowadays IoT devices still exhibit various shortcomings. For example, they lack secure default configurations and sufficient security configurability. They also lack rich behavioural descriptions, failing to list provided and required services. To answer this problem, we envision a future where IoT devices carry behavioural contracts and Fog nodes store network policies. One requirement is that contract consistency must be easy to prove. Moreover, contracts must be easy to verify against network policies. In this paper, we propose to combine the security-by-contract (S × C) paradigm with Fog computing to secure IoT devices. Following our previous work, first we formally define the pillars of our proposal. Then, by means of a running case study, we show that we can model communication flows and prevent information leaks. Last, we show that our contribution enables a holistic approach to IoT security, and that it can also prevent unexpected chains of events.

## 1. Introduction

Pervasive computing foresees a future where computing appears anytime and everywhere. To realise such a visionary paradigm, everyday objects have to embed two important features: Computational power and connectivity. The disruptive advent of Internet of Things (IoT) represents a first important step towards the realisation of such a pervasive computing vision. Not only can IoT devices form complex interconnected systems that exchange any kind of information, they can also connect to the Internet and interpret such information in a broader context. However, as for every technical result, some aspects of the IoT proved to be problematic. In particular, cybersecurity has been so far one of the biggest challenges for the IoT, as well as one of its most embarrassing failures [1].

Since the advent of IoT, the security research community has made an effort to raise awareness on the security challenges that the new paradigm brought. Some challenges, such as weak passwords or the lack of security updates, stem from best practices overlooked by manufacturers at the design phase [1]. Others, such as integration and automatic configuration, derive from the heterogeneous nature of the IoT itself [2].

As a matter of fact, IoT devices can have radically different goals. They range from smart plugs that turn appliances on/off, to fleets of devices that connect whole factories. These devices have different hardware and software requirements. They can equip different sensors, different communication protocols, and even different amounts of computing power. Heterogeneity is a strong point of IoT, and a weak one at the same time. On the one hand, the variety of devices enables to tailor solutions on specific requirements. On the other hand, achieving a robust and secure infrastructure is far harder than it used to be with traditional networks.

Moreover, many IoT devices are computationally limited and, aiming to overcome this limitation, manufacturers rely upon Cloud computing to undertake heavy workloads. The key problem with this two layer approach is that Cloud computing has not been designed for the volume, variety, and velocity of data that IoT generates. Some intrinsic drawbacks, such as unpredictable network latency and uncertain storage location, are particularly problematic for security-related IoT generated data.

To counterbalance these issues, Fog computing has been proposed as an extension to Cloud computing [3]. The key idea is to move critical services from the Cloud to the edge of the network, close to IoT devices. Typically installed in a network in the form of a dedicated device (i.e., the Fog node), the Fog provides a virtualized middle layer that sits between latency-sensitive applications and the Cloud. Its goal is to remedy some of the Cloud drawbacks by centralizing sensors data, undertaking time-bound tasks, and serving as a data traffic gatekeeper. Moreover, a Fog node is a trustworthy device that can store security-related data, such as network policies, and perform real-time policy enforcement techniques (e.g., enforcement based on traffic monitoring).

### 1.1. Contributions of the Paper

In this paper, we propose to combine security-by-contract (S × C) and Fog computing to strengthen IoT systems security. S × C have previously been applied for similar goals to various domains, such as mobile applications [4,5] and multi-application smart cards [6].

This work focuses on addressing one of the yet to be solved classes of threats concerning IoT systems, namely insufficient security configurability [2]. In a nutshell, current IoT devices offer little ways for configuring them according to to users’ necessities. For example, often IoT devices entirely neglect access control granularity, since they only provide the administrator login. In particular, the contributions of this paper are manifold:

- We provide a formal description of the pillars of the S × C approach, such as security rules, contracts, policies.
- We show how these concepts can be combined together to secure IoT systems.
- We define the notion of consistency for both contracts and policies.
- Based on the above concepts, we define several properties useful for the security of IoT systems, such as contract-policy matching and illegal information flow.
- We provide algorithmic versions of our formal definitions and methods, laying the foundation for a real-world implementation.
- We illustrate these concepts by means of a running case study, based on real-world smart home (E-care@home (http://ecareathome.se)).
- We show how S × C can successfully be integrated with Fog computing to secure IoT systems.
- We extensively discuss pros and cons of our approach in a threat model.

### 1.2. Paper Outline

This paper is organized as follows. In Section 2 we give an overview of IoT open security challenges. In Section 3 we present the general idea of the application of S × C to the IoT, and the running case study we use throughout our work. Then, in Section 4, Section 5 and Section 6 we define and describe the building blocks of our proposal, namely security rules, security contracts, and security policies. In Section 7 we show that our approach can also identify and prevent hidden communication paths stemming from physical communication channels. In Section 8 we summarize the potential threats to our Fog-based solution. Then, we discuss benefits and limitations of S × C in this context. In Section 9 we report the relevant related work. In Section 10 we discuss the ongoing development and the future work we have planned. Last, in Section 11 we wrap up our contribution.

## 2. IoT Smart Services: Background and Challenges

In this section, we give a brief bird’s-eye view on IoT specific security challenges, we summarize the related challenges, and then we clarify where our contribution sits with respect to this analysis.

In a recent paper, Zhou et al. [2] analysed the main security challenges (named features) that are characteristic of the IoT domain. They have identified eight different features, namely:

- interdependence
- diversity
- constrained
- myriad
- unattended
- intimacy
- mobile
- ubiquitous

In particular, under the umbrella of interdependence and ubiquitous features, the authors highlighted that the main threat for IoT devices is the default insecure configurations provided by the manufacturers, as well as the insufficient security configurability.

Over time, interactions between devices grow in complexity, while the human role in managing such interactions becomes less and less important. This is a complex problem from a security point of view. Manufacturers and researchers often focus on the robustness of the single device, or on the direct communications between devices. However, the devices’ interdependence makes hard to define specific security rules for every and each of them, often resulting in excessive privileges, granted preemptively. Moreover, the fact that IoT devices are ubiquitous and pervasive within modern society amplifies the aforementioned threats, particularly when IoT devices are manufactured with insecure default configurations.

At the time of writing, there are plenty of examples of insecure configurations and insufficient security configurability. Passwords are oftentimes hardcoded within the IoT devices and, if they can be changed, there are no entropy checks in place. There is no permission granularity, meaning that either the user logs in as admin or not at all, which impedes to develop layered security approaches which involve such devices. Other devices have services and connections ports open for everyone in the name of availability, in case those services are ever needed, and usually no monitoring over information flows are in place within IoT local networks.

Moreover, little self-configuration capabilities are present in current IoT devices. As pointed out by Athreya et al. [7], the cloud-centric approach to IoT management is likely to fail in the future, due to the scalability limitation that it entails. IoT devices need to be in charge of configuring and adapting themselves to the environment they belong to, without negatively affecting other devices and services. However, this is possible only if IoT devices declare to the environment their behavioural specifications.

Without behavioural descriptions, plugging an IoT device into an established infrastructure opens up to several uncertainties. What services does the device offer? Which ones does it need? Over which communication protocols? Does this device disrupt or disturb other running services? These are just few examples, out of many more.

Aiming to address this specific issue, Cisco proposed Manufacturer Usage Descriptions (MUD) [8], an authoritative identifier that allows manufacturers to describe the identity and the intended behaviour of the devices they produce. Intuitively, IoT devices carry an URI that redirects to a certified website, where the MUD file describing their behaviour is hosted. A trusted device within the private network, namely the MUD server, is responsible for reading the URI, pulling the MUD file, and granting privileges accordingly. Even though MUD has shown the way to tractable descriptions and configurations, in its current form it merely provides a device identification and a list of the addresses/ports used. In fact, MUD profiles are not rich enough to model complex behaviour, such as device-based permissions. Moreover, MUD profiles do not allow easy verification of device behaviour against the network security policy, nor against the intended behaviour of other devices in the same network. In this paper, we contribute to partially amending such shortcomings.

Last, in the aforementioned work, Zhou et al. [2] classified the body of literature about IoT security, and identified seven different classes of threats on which researchers have focused until today:

- privacy leaks
- insecure network communication/protocol
- vulnerable cloud/web service
- insecure mobile application
- vulnerable system/firmware
- insufficient security configurability
- other threats

Their analysis showed that the largest amount of research went into privacy leaks and insecure network communication issues, with different percentages for different scenarios. What stands out is that, although critical for the IoT, the insufficient configurability has still to be thoroughly addressed by the community. We believe that this research gap is due to the complex traits of this problem. Our effort aims at specifically addressing this key open challenge.

### Running Case Study: E-Care@Home

**Figure 1:**
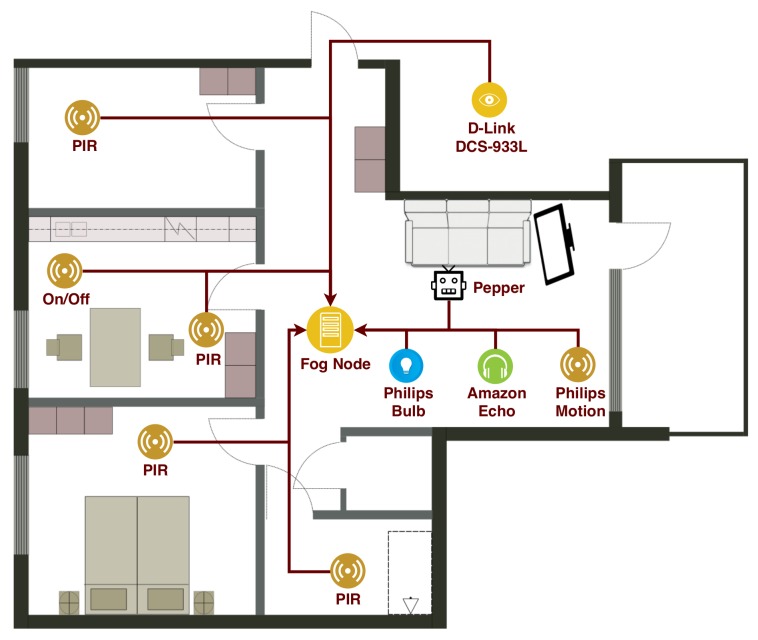
Ängen context-aware smart home, equipped with various sensors, like motion and pressure ones.

E-care@Home is a Swedish interdisciplinary distributed research environment that pulls together competences in artificial intelligence, semantic web, IoT and sensors for health [9]. Ängen, a real smart home composed by several IoT devices, was created within the E-care@home initiative (Figure 1 shows Ängen layout). Each room is equipped with PIR (passive infrared) motion sensors, running over different boards, such as Pycom WiPy 3.0 boards, and different boards running Contiki OS. Bed, sofa, and chairs use binary pressure switches to detect whether someone is sitting. In the kitchen, the oven has an on/off sensor. A smart light Philips Hue White v3.0, a Philips Hue Motion v1.0 motion sensor, and a smart speaker Amazon Echo v1.0 are installed in the living room. Moreover, in the living room we have Pepper, a humanoid robot from manufacturer SoftBank. For safety reasons, in the future there will be a D-Link DCS-933L security IoT camera right outside the facility door. Last, all devices are integrated through the E-care@Home middleware, from which a context reasoner extracts the necessary information. Currently, a simple laptop collects the ground truth, and infers information through a reasoner. In our future plans, captured by Figure 1, a Fog node will replace the laptop, and take on the necessary security tasks.

## 3. S × C Fundamentals

The S × C framework [4,5] is based on two key concepts, namely *contract* and *policy*.

A security contract (or simply contract) represents the specification of the behaviour of an IoT device for what concerns relevant security actions. An IoT device is equipped with a contract, and this contract has to be exhibited to the Fog node before being accepted to participate to the network.

A security policy (or simply policy) is instead a specification of the acceptable behaviour of the IoT devices a Fog node is responsible for, for what concerns relevant security actions.

It is clear that, to refine the concepts of contract and policy in the IoT domain, it is necessary to narrow down the meaning of relevant security actions. For the scope of this paper, we restrict the set of possible relevant security actions to the one of the possible interactions among IoT devices governed by a Fog node (for a broader set of actions we refer to Giaretta et al. [10]). In this particular setting, a contract describes which resources are necessary for the device to operate, and which resources the device provides to others. A policy declares what actions are allowed within that specific context, and what resources all the included devices need/provide.

**Figure 2:**
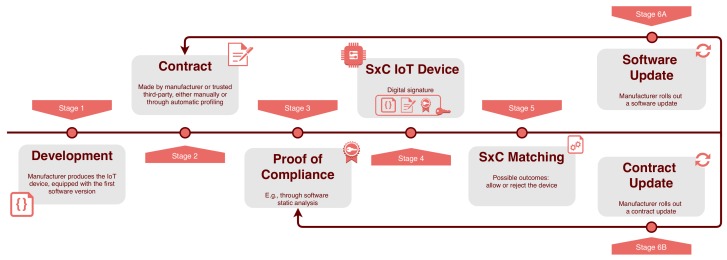
Security-by-contract (S×C) contract lifecycle. In Stage 4, the manufacturer digitally signs all the components, achieving software validation and non-repudiability: S × C provides semantics for digital signatures on an IoT code.

As shown in Figure 2, whenever an IoT device attempts to join the network its contract is parsed by the Fog node, which runs the matching algorithm and decides if the IoT device is compatible with the network policy, or not. Besides, if the device contract or its software are updated, the matching process re-starts, to ensure that the device is still compatible with the network policy.

### 3.1. Who Writes Contracts and Policies?

A question that naturally comes when we talk about contracts and policies is who are the actors that define them.

With regards to contracts, ideally they should be defined by the company manufacturing the IoT devices, for two main reasons. First, the user should have the assurance that, whenever he buys a new device and plugs it into a networked environment, right away the device is going to do exactly what it claims, without interfering with existing services. To this aim, the manufacturer can equip the device with proof-of-compliance (PoC), which binds the IoT software to the contract (proving that the software is compliant with the contract). This is a fundamental component of the S × C approach that, for space limitation, we do not touch on in this paper (more details in [10] and S × C literature). Second, it would be much easier for the manufacturer to create complete contracts from the beginning, instead of trying to define later a contract through reverse-engineering approaches which might leave out some hidden behaviour. Besides, this should put some pressure on manufacturers, forcing them to implement healthy lifecycles for their products.

However, this is not achievable for already-produced IoT devices. In this case, it is possible to extract (possibly incomplete) contracts through profiling routines. A device can be sandboxed and the consequent analysis can be performed over its traffic, in order to figure out its observed behaviour. A similar approach has been used with MUD policies by Hamza et al. [11], for extracting MUD profiles from legacy IoT devices. We also foresee that contracts can be customized at the time of first booting. As an example, when a smart light is installed it might ask which smartphone will be controlling it, through the manufacturer app.

On the other hand, policies are more complex than contracts and their sources might be quite different. For example, policies could be handed out by smart home manufacturers, consumer protection organizations, security companies, or non-profit organizations (e.g., the Electronic Frontier Foundation), in the same way that virtual operating system (OS) boxes are distributed (https://www.osboxes.org/virtualbox-images/). Moreover, policies could be defined from scratch by the network administrator himself, or created using the aforementioned ones as templates.

### 3.2. Implications

Security solutions should be kept simple in order to be effective. Overcomplicated tasks are likely to drive users and administrators away from security, or to encourage dangerous shortcuts. With this in mind S × C aims to have as simple results as possible.

With respect to simple users, S × C is completely transparent. Users’ devices store their own contract and, at connection time, the Fog node is responsible for verifying them. In the case that the contract is compliant with the policy, the device will be simply accepted. On the contrary, if the contract violates the policy, the user will be notified that the device is not compliant with the network policies.

Regarding network administrators, a little overhead is required for them. Administrators are responsible for managing the network and its policies, and the S × C policy would be part of their responsibility. However, as shown throughout this paper, policies are simple security rules, relatively easy to write and edit for administrators. Moreover, as mentioned in Section 3.1, S × C policies and templates could be written by trusted third parties. It would be easy for an administrator to start with a template and edit it according to his network necessities.

Last, manufacturers are the most affected by the introduction of S × C. First, they have the responsibility for writing their products’ contracts. Second, they write the PoC which formally binds code and contract. Moreover, whenever the manufacturer rolls out an update, it has to update both contract and PoC, accordingly. Even though this is a considerable overhead for a manufacturer, it can also be considered a great opportunity for improving product lifecycles. As a matter of fact, following an update with a consistency check between contract and actual behaviour not only protects the customers, it also helps manufacturers themselves to keep track of their own products. In Table 1 we summarise roles, actions, and implications.

## 4. S × C for IoT: Security Rules

In this section, we define the building blocks of our proposal. We start by describing how we refer to a device and its services. Then we define the concept of security rule.

**Definition 1**  ((IoT device))**.** *A device* D *is a well formed composition of a device name* d *and a manufacturer of the device* M, *expressed as* M.d.

**Definition 2**  (Service)**.** *A service* S *provided by an IoT device* D *=* M.d *is a well formed composition of a service name* s, *the servicing IoT device name* d *and the manufacturer of the device* M*. As a result,* S *is expressed as* M.d.s.

**Example 1**  (IoT device A)**.** *Referring to the camera* D-Link.933L, *previously mentioned in Section 2, this device provides two different services. The first service is called* SetDayNight, *and it is responsible for switching on/off the device LED; the second service is* SetSystemMotion, *which is responsible for enabling and disabling the integrated motion sensor. In terms of an S × C contract, we would refer to these two services as* D-Link.933L.SetDayNight *and* D-Link.933L.SetSystemMotion, *respectively.*

**Example 2**  (IoT device B)**.** *The device* Philips.HueWhite *we defined in Section 2 provides three different services called* On*,* Bri, *and* Hue. On *is used to remotely turn on/off the light, whereas* Bri *changes the brightness, and* Hue *changes the hue, according to the user preferences. Following our formalization, in a contract we would refer to these services as* Philips.HueWhite.On*,* Philips.HueWhite.Bri, *and* Philips.HueWhite.Hue, *respectively.*

**Definition 3**  (Domain)**.** *A domain* Dom *is a non-empty string identifying the context (in terms of network domain, such as a local area network (LAN)) where a security rule applies.*

### 4.1. Security Rule

**Definition 4**  (Security rule)**.** *A security rule (or simply, rule)* R *is a 5-tuple represented by the fields listed in Table 2.*

From now on, we will use the notation R[D] to denote the device D of a security rule R. Analogously, R[Dom], R[Shares], R[Provides], and R[Requires] denote the related fields of a rule R.

**Example 3**  (Security rule R${}_{A1}$). *The IoT camera surveillance* D-Link.933L *allows the owner to enable/disable the camera LED from his smartphone, over any domain. As mentioned in Section 3.1, we depict that, for example, at deployment time a smartphone can be chosen as a main controlling device. In order to express this, the resulting security rule would look as shown in Table 3.*

**Example 4**  (Security rule R${}_{A2}$)**.** *Let us now suppose that the IoT camera surveillance* D-Link.933L *enables the owner to access a* Philips.HueWhite *device, and turn on the lightbulb whenever the internal motion sensor of the camera detects a movement. Therefore, the camera requires service* On*, but it does not want to provide any service to other devices. In order to express this, the resulting security rule would look as shown in Table 4.*

**Example 5**  (Security rule R${}_{B1}$)**.** *The lightbulb* Philips.HueWhite *provides three different services (namely* On*,* Bri, *and* Hue*) to every* Philips *device. Moreover, the device requires a motion sensor* HueMotion *(produced by the same manufacturer,* Philips *), which provides service* Presence *and enables to switch on/off the lights, depending on the presence of people in the room. Last,* HueWhite *is designed to communicate only within the LAN. A security rule that expresses these requirements is shown in Table 5.*

### 4.2. Well Formed and Core Security Rules

In order to avoid malformed security rules, we introduce a notion of well formed security rule. Intuitively, a security rule R concerning a device D is well formed if the rule specifies which IoT devices can use the services provided by D. Since these devices are listed in R[Shares], we have that R[Shares] must not be empty in case D provides some services in R (i.e., R[Provides] is not empty). Note that this has several meanings: R[Shares] could include a specific list of devices, or whatever device of one or more manufacturers, or in general whatever device of whatever manufacturer. However, R[Shares] must not be empty if D provides some services.

**Definition 5**  (Well formed security rule)**.** *A security rule* R *is said to be well formed if and only if the following condition holds: If* R*[*Provides*] is not empty, then* R*[*Shares*] must not be empty.*

In Algorithm 1, we provide a pseudo-algorithm that verifies if a rule is well-formed and returns true/false, accordingly.

**Algorithm 1:** IsWellFormedRule Function **Require:** R **Ensure:**
*TrueorFalse*   **if**
R[Provides]≠∅
**and**
R[Shares]=∅
**then**   **return**
*False*   **end if**   **return**
*True*

**Example 6**  (Well formed security rule)**.** *The security rule defined in Example 4 is an example of a well formed security rule.*

**Example 7**  (Malformed security rule)**.** *Let us assume that we modify the security rule defined in Example 4, Table 4, and that we change both the* Provides *and* Requires *fields. Such modifications are shown in Table 6.*

We would obtain a malformed security rule, according to Definition 5. In particular, rule R${}_{\mathrm{MA}}$ claims that the device provides the SetDayNight service. However, R${}_{\mathrm{MA}}$[Shares] is empty, which violates Definition 5.

Ensuring that a security rule is well formed is necessary, but not sufficient, to achieve consistency among security rules. Intuitively, that is because two rules can be well formed with respect to themselves, but they could contradict each other. Thus, we want to capture the property that no rule concerning a device restricts another rule concerning the same device. Here restricts means that the rule provides less or the same number of services to the same set, or a subset, of devices. This concept of core rule will form the basis of the notion of consistent security contract in Section 5, where a set of rules represents the contract of a device.

**Definition 6**  (Core rule)**.** *Given a set of security rules* Set *= {*R${}_{1}$, *…,* R${}_{m}$*}, m>0, a rule* R ∈ Set *is said to be core with respect to* Set, *if and only if the following condition holds:*

R ∈ Set *is* core *iff* ∄ R${}_{★}$ ∈ Set*: (*R*[*D*] =* R${}_{★}$*[*D*])* ∧ *(*R*[*Dom*] =* R${}_{★}$*[*Dom*])* ∧ R${}_{★}$*[*Shares*] ≠∅∧ (*R${}_{★}$*[*Shares*] ⊆* R *[*Shares*]) ∧ (*R${}_{★}$*[*Provides*]* ⊆ R*[*Provides*]).*

In Algorithm 2, we provide a pseudo-algorithm that verifies if a rule is core and returns true/false, accordingly.

**Algorithm 2:** IsCoreRule Function **Require:** R, Set **Ensure:**
TrueorFalse  **for**
$\forall \;R_{★}$ ≠R
∈SET
**do**   **if** R[D
$]=R_{★}$[D]
**and** R [DOM$]=R_{★}$[DOM]
**then**    provideFlag←True, sharesFlag←True    **if** R_★_
[SHARES]=∅
**then**     sharesFlag←False    
**else**     **for** $\forall \;D_{∆}$
$\in R_{★}$
[SHARES]
**do**      
**if** $D_{∆}\notin R$
[SHARES]
**then**       
SharesFlag←False     **end if**    **end for**   **end if**   **for** $\forall \;S_{∆}$
$\in R_{★}$
[PROVIDES]
**do**    
**if** $S_{∆}\notin R$
[PROVIDES] **then**     
provideFlag←False    
**end if**   
**end for**   
**if** provideFlag=True
**and**
sharesFlag=True **then**    
**return** False   **end if**  **end if** **end for** **return** True

## 5. S × C for IoT: Security Contract

In the previous section we described the building blocks of our formalization. Next, we need to define security contracts. Informally, a security contract of an IoT device is a non-empty set of security rules describing the security behaviour of the device. Formally:

**Definition 7**  (Security contract)**.** *A security contract (or simply contract)* C${}_{D_{}}$ *of an IoT device* D *is a non-empty and non-ordered set of security rules concerning the device* D, *such that:*

C_D_ = *{*R_1_,…, R_n_*}*

*where n > 0 and ∀ i ≠ j:* R${}_{i}$*[D] =* R${}_{j}$ *[D],* R${}_{i}$ *≠* R${}_{j}$.

**Example 8**  (Security contract **C${}_{A}$**)**.** *An IoT camera surveillance enables Luke to enable/disable the camera LED from his smartphone over any domain, through the service* SetDayNight*. However, it enables him to access the administration panel only from within the network. For this to work, we need the* 933L *camera to have the contract in Table 7. Without considering security vulnerabilities resulting from vulnerable embedded software, this contract can easily prevent potential stalking episodes, which are more probable with currently manufactured IP cameras [12].*

**Example 9**  (Security contract **C${}_{B}$**)**.** *In this example, let us assume that* Philips.HueWhite *exposes rule* R${}_{B1}$ *which provides to* Philips *devices within the LAN domain three different services, namely* On*,* Bri, *and* Hue*. Moreover, the device requires service* Philips.HueMotion.Presence *for switching on/off the light, based on the presence of people in the room. Last,* HueWhite *exposes a second rule* R${}_{B2}$, *which provides access to services* On *and* Bri *to any device, within the LAN domain. A security rule that express these requirements is shown in Table 8.*

**Figure 3:**
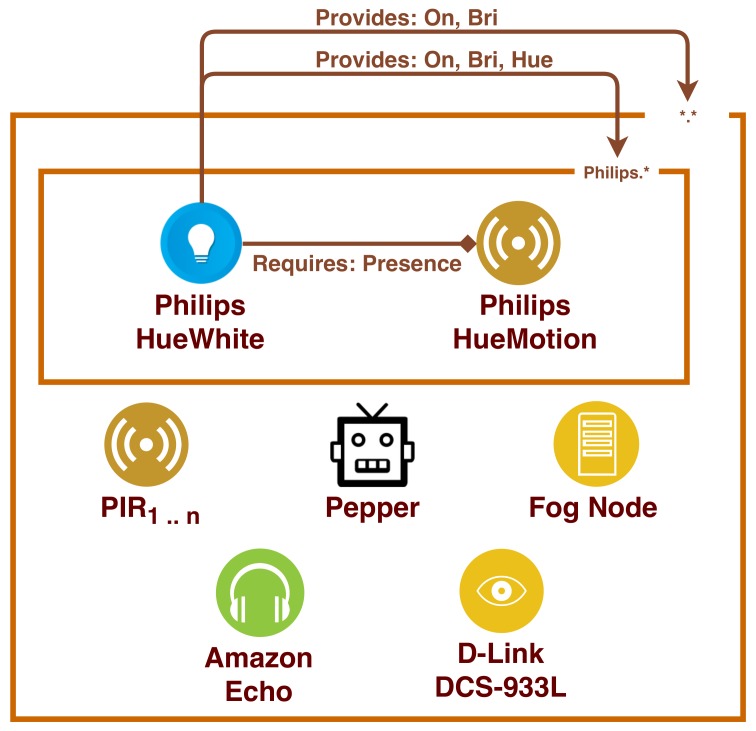
Security contract C${}_{B}$. Philips.HueWhite shares services On and Bri with all the devices in the LAN (e.g., with Amazon Echo). Service Hue is shared only with Philips devices, which means that in this scenario only Philips.HueMotion is allowed to use it. Moreover, Philips.HueWhite requires from Philips.HueMotion the service Presence.

Within the context of the Ängen smarthome (described previously in Figure 1), let us suppose that the device Philips.HueWhite is equipped with Contract C${}_{B}$. In Figure 3 we show the interactions that Philips.HueWhite would perform with the other devices, according to the behaviour defined in its contract. In particular, the figure shows that the only device that benefits from service Philips.HueWhite.Hue is Philips.HueMotion.

The next key concept we want to express in our framework is the consistent contract concept. Consistency is seen here as a quality check of a contract. First, we require all the rules in a consistent contract to be well formed. Second, we want that all the rules in the contract are core, to be sure that no rule in the contract restricts another rule in the same contract. As a result, a consistent contract is a set of well formed and core rules.

**Definition 8**  (Consistent security contract)**.** *A security contract* C${}_{D_{}}$ *of a device* D *is said to be* consistent *if and only if the following two conditions hold:*

*1.*  ∀ R ∈ C${}_{D_{}}$ *,* R *is well formed*
*2.*  ∀ R ∈ C${}_{D_{}}$ *,* R *is core in* C${}_{D_{}}$

In Algorithm 3, we provide a pseudo-algorithm that verifies if a contract is consistent and returns true/false accordingly.

**Algorithm 3:** IsConsistentContract Function **Require:** C_D_ **Ensure:** TrueorFalse  **for**
∀R
∈C_D_
**do**   
**if** IsWellFormedRule(R) =False
**then**    **return** False   **end if**
  **end for**  **for** ∀R ∈C_D_
**do**   
**if** IsCoreRule(R, C_D_) =False
**then**    **return** False   **end if**  **end for**  **return**  True

**Example 10**  (Consistent security contract **C${}_{B}$**)**.** *The security contract described in Example 9 is an example of a consistent security contract, with respect to Definition 8.*

**Example 11**  (Inconsistent security contract **C${}_{\mathrm{IB}}$**)**.** *Let us assume that we modify the security contract described in Example 9. In particular, let us remove both* Bri *and* Hue *from rule* R${}_{B1}$ *(obtaining rule* R${}_{B3}$ *). The contract shown in Table 9 formalizes our goal.*

*In this case, the resulting contract* C${}_{\mathrm{IB}}$ *contradicts Definition 8, because rule* R${}_{B3}$ *restricts rule* R${}_{B2}$ *. In particular, less services are provided to less devices in the same domain (i.e.,* R${}_{B3}$ *[* Provides *] ⊆* R${}_{B2}$ *[* Provides *] ∧* R${}_{B3}$ *[* Shares *] ⊆* R${}_{B2}$ *[* Shares *]).*

## 6. S × C for IoT: Security Policy

Building on top of the previous sections, in this section we describe security policies. Informally, a security policy of a Fog node is a non-empty set of security rules describing the allowed security behaviour of the devices for which the Fog node is responsible. Formally:

**Definition 9**  (Security policy)**.** *A security policy (or simply policy)* P${}_{F_{}}$ *of a Fog node* F *is a non-empty and non-ordered set of security rules, such that:*

$$P_{F_{}}=\left\{R_{1},\ldots ,R_{m}\right\},\mathrm{where}\;m>0\;\mathrm{and}\;R_{i}\neq R_{j},i\neq j.$$

From a practical perspective, the security rules within a policy P${}_{F_{}}$ can derive from two different sources: The contracts C${}_{D_{1}}$, …, C${}_{D_{n}}$ of the IoT devices the Fog node F is responsible for, and a number of additional contracts C${}_{D_{1}}^{A_{}}$, …, C${}_{D_{p}}^{A_{}}$ defined by the administrator A, which apply to the respective devices D${}_{1}$, …, D${}_{p}$. This set of additional contracts can be empty, in case the administrator does not define any specific security rule. As a result, a policy of a Fog node F can be seen as union of the contracts of the IoT devices and the contracts defined by the administrator of the Fog node.


P${}_{F_{}}$ = C${}_{D_{1}}$, …, C${}_{D_{n}}$, C${}_{D_{1}}^{A_{}}$, …, C${}_{D_{p}}^{A_{}}$

**Example 12**  (Security policy **P${}_{A}$**)**.** *In the example we show in Table 10, a policy is a composition of three different rules, one from device* D-Link.933L, *and two from device* Philips.HueWhite*. It is worth noticing that a security policy can have rules for different devices, as long as these rules do not contradict each other (we will better clarify this point in the rest of the section).*

**Example 13**  (Security policy **P${}_{B}$**)**.** *In this second example, shown in Table 11, let us suppose that the administrator wants to add a rule to his security policy. This rule (namely, rule* R${}_{\mathrm{Admin}1}$ *) allows every device from manufacturer* Philips *to use service* D-Link.933L.SetDayNight *over the Internet.*

Analogously to the security contract case, what we want to capture in our S × C framework is the concept of consistent security policy. Since a policy includes rules of contracts from different devices, the notion of consistency given in the contract case is not enough. This is because the contracts of two different devices might lead to a leak of information (or illegal information exchange). This is the case of a device D${}_{1}$ that (1) requires some services provided by a device D${}_{2}$, but (2) D${}_{2}$ cannot share these services to D${}_{1}$ because it would lead to information leakage (due to the fact that D${}_{1}$ can communicate with devices that D${}_{2}$ refuses to communicate with). In the following, we formalise (1) by means of the concept of direct communication among two IoT devices, and the consequent concept of allowed information flow. Then, we formalise (2) by means of the concept of forbidden information flow. We will then combine these concepts to define the notion of illegal information exchange, which is what we want to avoid in a (consistent) policy.

**Definition 10**  (Direct Communication)**.** *Given a device* D${}_{1}$ *with consistent contract* C${}_{D_{1}}$ *and a device* D${}_{2}$ *with consistent contract* C${}_{D_{2}}$, *we say that* D${}_{1}$ *directly communicates with* D${}_{2}$, *denoted* D${}_{1}$ *⇝* D${}_{2}$, *if* ∃ R${}_{★}$ ∈ C${}_{D_{1}}$ *,* R${}_{\circ }$ ∈ C${}_{D_{2}}$ *:* R${}_{★}$*[*Requires*] ∩* R${}_{\circ }$ *[*Provides*]* ≠∅.

In Algorithm 4, we provide a pseudo-algorithm that verifies if two devices directly communicate.

**Definition 11**  (Allowed direct communication). *Given a device* D${}_{1}$ *with consistent contract* C${}_{D_{1}}$ *and a device* D${}_{2}$ *with consistent contract* C${}_{D_{2}}$, *such that* D${}_{1}$ *⇝* D${}_{2}$, *we say that* D${}_{1}$ *is allowed to directly communicate with* D${}_{2}$, *denoted* D${}_{1}$ *→* D${}_{2}$, *if ∀* R${}_{★}$ ∈ C${}_{D_{1}}$ *,* R${}_{\circ }$ ∈ C${}_{D_{2}}$ *:* R${}_{★}$ *[*Requires*] ∩* R${}_{\circ }$ *[*Provides*] ≠∅, we have* D${}_{1}$ ∈ R${}_{\circ }$ *[*Shares*].*

**Algorithm 4:** DirectCommunication Function **Require:**
CD${}_{1}$,CD${}_{2}$ **Ensure:**
TrueorFalse  **for** $\forall \;R_{★}$ ∈CD${}_{1}$ **do**   **for** $\forall \;R_{\circ }$ ∈CD${}_{2}$ **do**    
**if** R_★_ [DOM$]\;\cap \;R_{\circ }$ [DOM]    
**and** R_★_$[REQUIRES]\cap R_{\circ }$ [PROVIDES]≠∅ **then**     **return** True    **end if**   **end for**  **end for**  **return** False

In Algorithm 5, we provide a pseudo-algorithm that verifies if two devices are allowed to directly communicate.

**Definition 12**  (Allowed information flow)**.** *Given a device* D${}_{1}$ *with consistent contract* C${}_{D_{1}}$ *and a device* D${}_{2}$ *with consistent contract* C${}_{D_{2}}$, *such that* D${}_{1}$ *→* D${}_{2}$, *there is an allowed information flow between* D${}_{1}$ *and* D${}_{2}$, *denoted* D${}_{1}$ *$\vec{ok}$* D${}_{2}$, *if ∀* R${}_{★}$ ∈ C${}_{D_{1}}$ *,* R${}_{\circ }$ ∈ C${}_{D_{2}}$ *:* R${}_{★}$ *[*Requires*] ∩* R${}_{\circ }$ *[*Provides*] ≠∅, we have* R${}_{★}$ *[*Shares*] ⊆* R${}_{\circ }$ *[*Shares*].*

**Algorithm 5:** AllowedDirectCommunication Function **Require:**CD${}_{1}$,CD${}_{2}$ **Ensure:** TrueorFalse  **if** DirectCommunication(C${}_{D_{1}}$, C${}_{D_{2}}$) =False **then**   **return** False  **end if**  **for** $\forall \;R_{★}$ ∈C D${}_{1}$ **do**   **for** $\forall \;R_{\circ }$
∈CD${}_{2}$
**do**    
**if** R_★_[DOM] $\cap \;R_{\circ }$ [DOM]
    **and** D_1_ $\notin \;R_{\circ }$ [SHARES]    **and** 
R_★_$[REQUIRES]\;\cap R_{\circ }$ [PROVIDES] ≠∅ **then**
     
**return** False    **end if**   **end for**  **end for**  **return** True

**Definition 13**  (Forbidden information flow)**.** *Given a device* D${}_{1}$ *with consistent contract* C${}_{D_{1}}$ *and a device* D${}_{2}$ *with consistent contract* C${}_{D_{2}}$, *such that* D${}_{1}$ *→* D${}_{2}$, *there is a forbidden information flow between* D${}_{1}$ *and* D${}_{2}$, *denoted* D${}_{1}$ *$\vec{no}$* D${}_{2}$, *if* ∃ R${}_{★}$ ∈ C${}_{D_{1}}$ *,* R${}_{\circ }$ ∈ C${}_{D_{2}}$ *:* R${}_{★}$ *[*Requires*] ∩* R${}_{\circ }$ *[*Provides*] ≠∅∧* R${}_{★}$ *[*Shares*] ⊈* R${}_{\circ }$ *[*Shares*].*

In Algorithm 6, we provide a pseudo-algorithm that verifies if a forbidden information flow exists between two devices.

**Algorithm 6:** ForbiddenInformationFlow Function **Require:** C_D_${}_{1}$,CD${}_{2}$ **Ensure:** TrueorFalse  **if** AllowedDirectCommunication(C${}_{D_{1}}$, C${}_{D_{2}}$) =False
**then**   **return** False  **end if**  **for** $\forall \;R_{★}$ ∈ CD${}_{1}$ **do**   **for** $\forall \;R_{\circ }$ ∈ CD${}_{2}$
**do**   **if** R_★_[DOM] $\cap \;R_{\circ }$[DOM]    **and**
R_★_$[SHARES]\;\subseteq R_{\circ }$[SHARES]    **and** R_★_$[REQUIRES]\;\cap \;R_{\circ }$[PROVIDES] ≠∅ **then**     
**return** True    **end if**   **end for**  **end for**  **return** False

**Definition 14**  (Illegal information exchange)**.** *Given a consistent contract* C${}_{D_{1}}$ *of a device* D${}_{1}$ *and a security policy* P${}_{F_{}}$ *of a Fog node* F, *there is an illegal information exchange, denoted* C${}_{D_{1}}$ *⇏* P${}_{F_{}}$, *if there exists a contract* C${}_{D_{2}}$ ∈ P${}_{F_{}}$ *such that (* D${}_{1}$ *$\vec{no}$* D${}_{2}$ *) ∨ (* D${}_{2}$ *$\vec{no}$* D${}_{1}$ *).*

In Algorithm 7, we provide a pseudo-algorithm that verifies if two devices generate an illegal information exchange.

**Algorithm 7:** IllegalInformationExchange Function **Require:**  C_D_${}_{1}$,P_F_ **Ensure:** TrueorFalse  **for** ∀CD${}_{2}$ ∈ P_F_ **do**   **if** ForbiddenInformationFlow(C${}_{D_{1}}$, C${}_{D_{2}}$) **or**   ForbiddenInformationFlow(C${}_{D_{2}}$, C${}_{D_{1}}$) **then**    **return** True   **end if**  **end for**  **return** False

**Example 14**  (Illegal information exchange)**.** *For the sake of simplicity, in Table 12 we suppose that* C${}_{D_{1}}$ *and* C${}_{D_{2}}$ ∈ P${}_{F}$ *are composed of a single rule, respectively.*

*According to Definition 14, this scenario would cause an illegal information exchange. First, we can verify that* C${}_{D_{2}}$*[*Requires*] ∩*C${}_{D_{1}}$*[*Provides*] =∅, therefore there is no allowed direct communication*D${}_{2}$*→*D${}_{1}$*and the second condition of the illegal information exchange (*D${}_{2}$*$\vec{no}$*D${}_{1}$*) can be ignored.*

*Second, we can verify that*D${}_{1}$*→*D${}_{2}$, *since that*C${}_{D_{1}}$*[*Requires*] ∩*C${}_{D_{2}}$*[*Provides*] =*On*(therefore, ≠∅), and that*D-Link.933L ∈ C${}_{D_{2}}$ *[*Shares *]. However,* C${}_{D_{1}}$ *[*Shares*] (= {*Apple.LukePhone*}) ⊈*C${}_{D_{2}}$ *[*Shares*] (= {*D-Link.933L*,* Philips.HueWhite*}). Therefore, we have* D${}_{1}$ *$\vec{no}$* D${}_{2}$ *and an illegal information exchange.*

**Figure 4:**
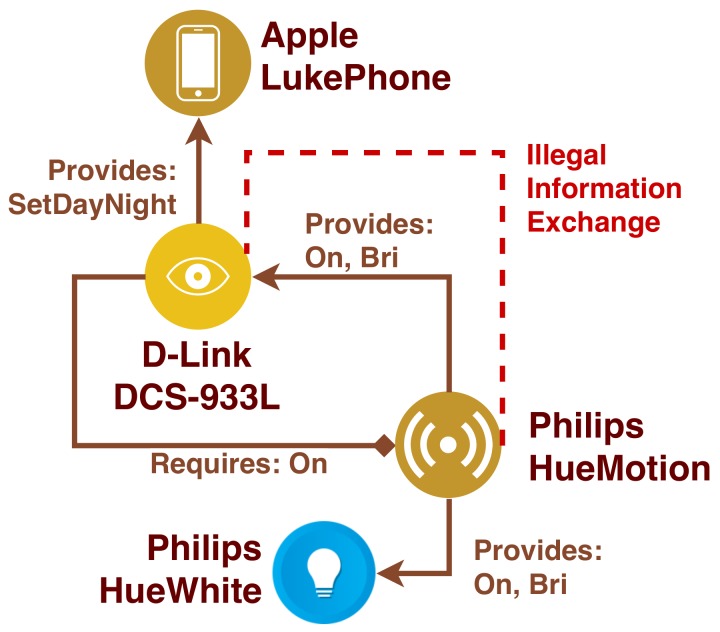
*Illegal information exchange*C${}_{D_{1}}$*⇏*P${}_{F_{}}$. D-Link.933L *does not want to share any service with*Apple.LukePhone *(i.e., there are no outgoing arrows), but*Philips.HueMotion*can potentially act as a bridge between the two devices. This might lead to critical information leak, therefore it has to be prevented.*

*Looking at Figure 4, the outgoing arrows for*D-Link.933L *and* Philips.HueMotion *clearly show that it an illegal information exchange exists between the two devices. In particular,* D-Link.933L *shares a set of services with* Philips.HueMotion *and* Philips. HueMotion *shares another set with* Apple.LukePhone*. However,* D-Link.933L *does not share any service with* Apple.LukePhone *and this flow might leak unintended information. Therefore, an illegal information exchange error is raised here.*

**Definition 15**  (Consistent security policy). *A security policy* P${}_{F_{}}$ *of a Fog node* F *is said to be consistent if and only if the following conditions hold:*

*1.*  ∀ R *∈* P${}_{F_{}}$*,* R *is well formed*
*2.*  ∀ R ∈ P${}_{F_{}}$*,* R *is core in* P${}_{F_{}}$
*3.*  *∄*C${}_{D_{}}$ ∈ P${}_{F_{}}$*,* C${}_{D_{}}$ ⇏ P${}_{F_{}}$
*4.*  *∄*C${}_{D_{}}^{A_{}}$ ∈ P${}_{F_{}}$ *,* C${}_{D_{}}^{A_{}}$ ⇏ P${}_{F_{}}$

In Algorithm 8, we provide a pseudo-algorithm that verifies if a policy is consistent.

**Example 15**  (Consistent security policy **P${}_{\mathrm{IA}}$**)**.** *The security policy described in Example 12 is an example of a consistent security policy, with respect to our own Definition 15.*

**Algorithm 8:** IsConsistentPolicy Function **Require:** *P*F **Ensure:** TrueorFalse  **for** ∀R ∈P_F_ **do**   **if** IsWellFormedRule(R) =False **then**    **return** False   **end if**  **end for**  **for** ∀R ∈P_F_ **do**   **if** IsCoreRule(R, P${}_{F_{}}$) =False **then**    **return** False   **end if**  **end for**  **for** ∀CD ∈ P_F_ **do**   **if** IllegalInformationExchange(C${}_{D_{}}$, P${}_{F_{}}$) **then**    **return** False   **end if**  **end for**  **for** ∀
C${}_{D_{}}^{A_{}}$ ∈ P_F_ **do**   **if** IllegalInformationExchange(C${}_{D_{}}^{A_{}}$, P${}_{F_{}}$) **then**    **return** False   **end if**  **end for**  **return** True

**Example 16**  (Inconsistent security policy **P${}_{\mathrm{IB}}$**)**.** *Let us suppose that the administrator modifies the policy previously defined in Example 13. In particular, let us suppose that the administrator substitutes rule* R${}_{\mathrm{Admin}1}$ *with rule* R${}_{\mathrm{Admin}2}$, *enabling* Philips.HueWhite *to provide every* Philips *device three services, as shown in Table 13. In this case, the policy would not be consistent, since rule*R${}_{B2}$ *would restrict Rule* R${}_{\mathrm{Admin}2}$*. In particular, rule* R${}_{\mathrm{Admin}2}$ *would not be core, violating the fourth condition defined in Definition 15.*

The last concept we have to define concerns the matching between a contract of a device and the policy of a Fog node. This is the core idea behind the S × C approach: A device is accepted in a network of devices governed by a Fog node if and only if the contract of the device matches the policy of the Fog node.

**Definition 16**  (Contract–policy matching)**.** *Given a contract* C${}_{D_{}}$ *of a device* D *and a consistent policy* P${}_{F_{}}$ *of a Fog node* F, *we say that* C${}_{D_{}}$ *matches* P${}_{F_{}}$ *if* P${}_{F_{}}^{{}^{\prime }}$ *=* P${}_{F_{}}$ *∪* C${}_{D_{}}$ *is consistent.*

In Algorithm 9 we show the pseudo-code for our matching algorithm. All the rules from the contract C${}_{D_{}}$ are added one by one to a temporary policy P${}_{F_{}}^{{}^{\prime }}$. Then, the consistency of P${}_{F_{}}^{{}^{\prime }}$ is verified and the match can return either true or false.

**Algorithm 9:** Matching Function **Require:**  *P*_F_,C_D_ **Ensure:** TrueorFalse  P${}_{F_{}}^{{}^{\prime }}$←P_F_  **for** ∀R ∈ C_D_ **do**   P${}_{F_{}}^{{}^{\prime }}$←P${}_{F_{}}^{{}^{\prime }}$+R  **end for**  **if** IsConsistentPolicy(P${}_{F_{}}^{{}^{\prime }}$) =True
**then**   **return** True  **end if**  **return** False


## 7. Jumping the Air Gap

Agadakos et al. [13] have recently highlighted that one of the key issues with the IoT is that security is solely considered on device and device-to-device levels. They claim that, given the complexity of interactions that happen in the IoT, the approach should be much more holistic. We totally agree with their view.

In this section, we show that our approach can nicely model the case studies proposed by Agadakos et al., as well as prevent the issue of unexpected chains of events they depicted.

### 7.1. Hidden Paths

**Figure 5:**
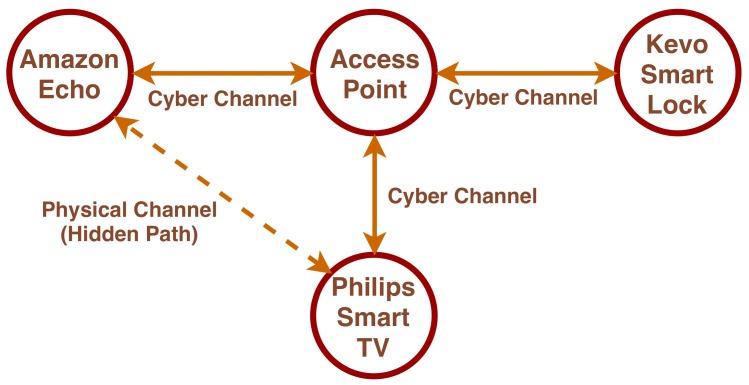
The topological map for the use case defined by [13].

In this scenario, there are communication paths, fully or partially composed of physical interactions that are hidden. As an example, Agadakos et al. consider a typical smart living room equipped with an Amazon Echo device and a Philips Smart TV, and assume that no direct cyber channels exist between the two, as shown in Figure 5. However, since the smart TV produces sound outputs, and the Amazon Echo does not have any protection over its input vocal channel (such as vocal identification), a hidden communication path exists between the two devices.

**Example 17**  (Security contract**C${}_{\mathrm{Echo}1}$**)**.** *In our proposal, a hypothetical contract carried by Amazon Echo would explicitly state that the device has a vocal service (as an example, let us call it VocalInput) which allows anyone to communicate with it, within the LAN domain. Enforcing explicit declarations about services, as shown in Table 14, would help to identify potential hidden paths, if not to avoid them completely.*

Another issue that has been raised in the past about vocal assistants is that the vocal perimeter can easily extend outside the smart home (https://techcrunch.com/2017/05/23/alexa-dont-talk-to-strangers/). A person walking by an open window can easily talk out loud and activate a vocal assistant placed in the living room. This situation can be easily captured by S × C contracts, as shown in the following Example 18.

**Example 18**  (Security contract **C${}_{\mathrm{Echo}2}$**)**.** *In order to model an extended vocal perimeter, a contract would simply have to state that the VocalInput function is reachable by anyone in any domain, meaning that anyone outside the LAN (human being or device) can activate the Amazon Echo without belonging to the same LAN. An example of this contract is shown in Table 15.*

The previous explicit contracts, namely C${}_{\mathrm{Echo}1}$ and C${}_{\mathrm{Echo}2}$, would help the administrator to have a grasp of potential attack vectors introduced by new devices and to setup proper policies accordingly.

**Example 19**  (Security policy **P${}_{\mathrm{FR}}$**)**.** *Let us suppose that a Federal Reserve building has in place policies that impede any kind of communication with the outside world, for safety reasons. In particular, the network administrator wants to avoid that a naive employee takes his brand new Amazon Echo to the office and plugs it in the network, exposing the building to external attacks. As we show in Table 16, with S × C it would be easy to avoid this scenario. Assuming that* R${}_{\mathrm{FR}1}$ *is already in place, when the Amazon Echo device is plugged in and shows its Contract* C${}_{\mathrm{Echo}2}$, *Rule* R${}_{E2}$ *is evaluated non-core within* P${}_{\mathrm{FR}}$ *(since it is restricted by rule* R${}_{\mathrm{FR}1}$*), and the Amazon Echo is not accepted. For the sake of clarity, in this example we wrote only the relevant rules for this discussion, but more rules could be part of this policy, as shown in previous sections.*

### 7.2. Security Degradation

Agadakos et al. identified another problem arising from combining IoT devices, namely the security degradation problem. This problem, similar to the confused deputy problem, states that a device which receives inputs over unauthenticated channels, but sends outputs over authenticated ones, can be tricked by malicious attackers into performing actions in their stead.

**Example 20**  (Security degradation as an illegal information exchange)**.** *In terms of our work, this problem is an instance of an illegal information flow. A Samsung Hub requires a service OpenClose from a Samsung Window Sensor, in order to alarm the user if the window is open. The Hub is powered by an OORT Smartplug, therefore the Hub contract declares that it shares its OnOff service with the Smartplug. Last, the Smartplug receives unauthenticated messages over a BLE channel so its contract states two things: That it shares all its services with anyone in the LAN domain, and that it requires the Samsung Hub OnOff service (to make explicit that it is capable of turning on/off the Hub). The resulting (inconsistent) security policy is shown in Table 17.*

*In particular, the reason why this policy is inconsistent is because there is an illegal information exchange*D${}_{\mathrm{Hub}}$*$\vec{no}$*D${}_{\mathrm{Sensor}}$, *according to Definition 14.*

*First, there is a*D${}_{\mathrm{Hub}}$*→*D${}_{\mathrm{Sensor}}$, *since*C${}_{D_{\mathrm{Hub}}}$*[*Requires*] ∩*C${}_{D_{\mathrm{Sensor}}}$*[*Provides*] =*OpenClose*(i.e., ≠∅), and*Samsung.Hub ∈ C${}_{D_{\mathrm{Sensor}}}$ *[* Shares *]. However,* D${}_{\mathrm{Hub}}$ *$\vec{no}$* D${}_{\mathrm{Sensor}}$ *is equally true. In particular,* C${}_{D_{\mathrm{Hub}}}$ *[* Shares *] (= {* OORT.Plug *}) ⊈* C${}_{D_{\mathrm{Sensor}}}$ *[* Shares *] (= {* Samsung.Hub *}), which entails an illegal information exchange* D${}_{\mathrm{Hub}}$ *$\vec{no}$* D${}_{\mathrm{Sensor}}$.

### 7.3. Transitions and States

As a last use case, in [13] the authors show that with their approach it is possible to model transitions and states, allowing them to identify security violations over time. In their example, there is a Roomba Vacuum that should never leave the house, and an OORT Smart Lock capable of closing/opening a garage door to the outside. Similar to Section 7.2, this issue is fuelled by the fact that the smart lock is allowed to communicate over BLE with any device, and that this is not formally declared to the network. However, if the OORT lock shows a contract similar to C${}_{\mathrm{OORT}\mathrm{Lock}}$ in Table 18, the administrator can have a clear view about the device characteristics, and a strong policy can easily refuse this device to join the network.

As we have previously stated in Section 7.1, enforcing devices to declare required and provided services considerably strengthens a network: Explicit behavioural descriptions allow for a much easier holistic view on the chain of events that can potentially happen in a complex IoT environment.

## 8. Threat Model

In this work, we have focused on two main threats affecting nowadays IoT systems (Zhou et al. [2]): Insufficient security configurability and, partially, insufficient security configurations. Of course, many other weaknesses can arise from IoT networks and it is out of the scope of this article to address all of them. As an example, with regards to the threats listed in Section 2, we do not address insecure network communications, nor vulnerable cloud/web services. Moreover, our solution partially covers other problems of the same list, as a side effect. Namely, we mitigate privacy leaks through the concept of illegal information exchange, and we mitigate vulnerable firmwares by means of signed proofs of compliance (PoCs).

**Figure 6:**
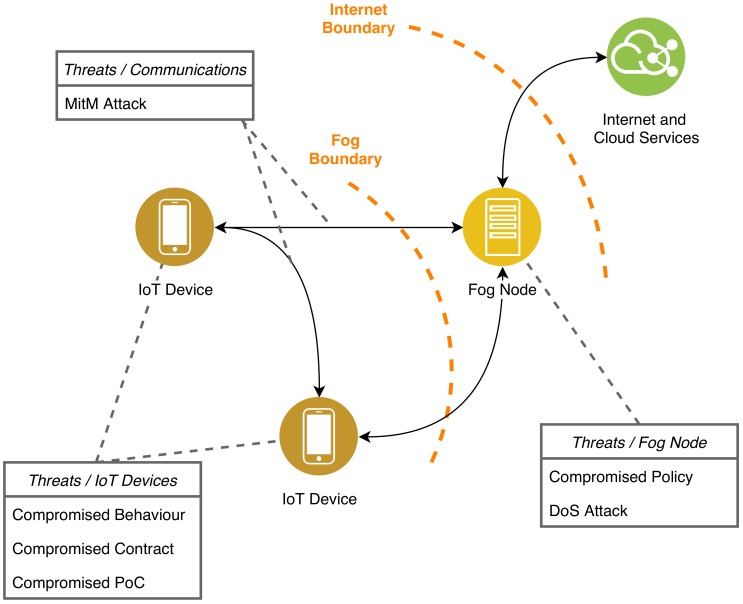
Graphical representation of the architecture threat model.

In order to have a better picture about the robustness of the S × C approach, in Figure 6 we present a threat model. In the picture we highlight the attacks that could be performed on the main actors of a Fog-based IoT system. The reader will notice that our threat model does not list each and every possible attack. We focus only on the most well known and disruptive ones. In the remainder of this section, we discuss the specific threats and the system resilience with/without S × C.

### 8.1. Threats to IoT Devices

Let us start from the threats to the IoT devices shown in Figure 6. As noted before, IoT devices currently sold on the market do not carry behavioural descriptions. Therefore, it is actually not clear and not simple to find out whether an IoT device has been tampered with or not.

With S × C, we equip IoT devices with a contract and a proof-of-compliance (PoC). The PoC is a proof, signed by the manufacturer and stored in the IoT device, which binds the onboard code to the contract. The PoC must be formally verifiable through an external validator. If the code or the contract change, due to a software update, the manufacturer has to issue and sign a new PoC. Thus, not only a malicious actor must create a new contract, in order to tamper with a device. He must also be able to forge a new PoC and sign it with the manufacturer credentials. If the PoC matches both the code and the contract, we say that the triplet <Code, Contract, PoC> is consistent. If the three elements do not match, the triplet is inconsistent.

Let us assume that a malicious actor tampers with the contract, but not with the PoC nor the code. As mentioned before, the PoC would not match with the contract and the code, thus the triplet would not be consistent. At installation time, the device can be rejected, but at runtime the Fog Node monitors only the device behaviour. Therefore, the Fog node can discover that the contract has changed only when the device behaves differently from the contract, stored in the policy. As soon as an unexpected behaviour emerges, the Fog node acts as if the contract have been updated and checks the triplet again. In this case, the Fog node finds out that the triplet is inconsistent and rejects the device.

As an example, if an attacker breaches into the Amazon Echo described in Table 14 and removes the service VocalInput, the triplet is not consistent any more. At installation time the Fog node rejects the IoT device or notifies the inconsistency to the administrator. At runtime the Fog node does not realize the breach, until the device behaves unexpectedly.

The situation is similar if the attacker tampers with the PoC, but does not modify the code nor the contract. At installation time the triples is inconsistent, so the device is rejected right away. If the PoC changes at runtime, the Fog node does not discover it, but no problems arise since that the device still behaves as expected (i.e., code and contract are unaltered). Legacy IoT devices do not carry contracts nor PoCs, therefore the aforementioned scenarios only apply to S × C.

Now, suppose that a malicious actor tampers with the code, but not with the PoC nor the contract. Again, this would create an inconsistent triplet. The Fog node would notice at runtime the discrepancy as soon as the tampered-with device behaves differently from its contract. However, a non-S × C architecture cannot identify this security threat, and it would allow the IoT device to join the network.

Last, suppose that a malicious attacker manipulates the entire triplet, so that it looks consistent. It is worth highlighting that the PoC must be signed by the manufacturer, hence forging a PoC is not an easy task. Without S × C the IoT device would join the network, and it would be easy for the attacker to perform malicious actions. Keeping in mind that the Fog node performs real-time monitoring, with S × C we have two different scenarios. In cases where the IoT device violates at run-time the network policy, the Fog node can identify the inconsistency and react. On the other hand, if the code has been tampered with in a way that it still complies with the policy, S × C cannot detect the breach.

In Table 19 we summarise these concepts, showing the benefits of S × C as well as this limitation.

We can better understand the last row of the S × C column in Table 19, if we take another point of view. IoT devices can receive updates over their lifecycle and their behaviour can change. This means we cannot reject IoT devices on the basis of inconsistency between current and past behaviour. We can only rely upon the triplet. Therefore, if the triplet is consistent and the PoC looks genuine, we cannot decide if the device underwent an honest update or a well-executed violation. Referring again to the contract in Table 14, let us assume that the attacker removes VocalInput and forges a consistent triplet <Behaviour, Contract, PoC>. In this case, there is no way to decide whether the contract modification was genuine or not.

Other threats might arise from misusing services, while formally complying with the security policy. For the sake of argument, we take the policy described in Table 17 and we show a consistent alternative in Table 20.

Let us suppose that an attacker violates Samsung.Hub. Without changing anything in the device, the attacker issues hundreds of commands to Samsung.Sensors.OpenClose. This is perfectly fine with the network security policy. However, this behaviour is clearly not as intended, and it might lead to serious damages to Samsung.Sensor. The problem resides in the very nature of declarative security approaches. If the framework is not powerful enough to describe fine-grained behaviour, policies cannot mark allowed actions nor forbidden ones.

### 8.2. Threats to Fog Node and Communication Channels

It is worth mentioning that our solution introduces some potential threats as a side effect. The main culprit is the Fog node. As for every centralized solution, single points of trust translate into single points of failure. When we entrust a Fog node with storing the network policy, we centralize critical tasks in a single actor. As a consequence, the Fog node is going to be a likely target for a malicious attacker.

Three main issues could arise. First, an attacker could aim to breach the Fog node and manipulate the policy. As an example, he could cut communications between two critical devices by means of a restrictive policy. On the opposite spectrum, the attacker might try to allow flows the administrator wanted to impede. In this case, the attacker can be more permissive than the administrator but this will not override the devices contracts. Thus, the attacker cannot force the devices to act differently from their intended behaviour. Third, the same attacker might decide to perform a DoS attack, aiming to disrupt the entire network.

In its current form, the S × C architecture we designed is prone to such attacks. However, there are a couple of potential solutions. One solution is replication, a common strategy for fault-tolerant systems. Indeed, introducing multiple instances of a Fog node within the network, we can mitigate some risks. Another solution would be to implement fallback routines in the IoT devices, and allow them to maintain communications over machine-to-machine (M2M) channels. In this scenario, the devices would bypass the compromised Fog node and negotiate directly, according to their own contracts.

Last, but not least, we would like to mention that a multitude of man-in-the-middle (MitM) attacks are possible on any networked systems. For the sake of simplicity, in our paper we assumed the channels to be secure and tamper-proof. However, it is good to keep in mind that many dangers can come from insecure communication flows. In Table 21, we summarise the issues introduced by a single Fog node.

## 9. Related Work

In recent years, IoT security has been largely discussed. Matheu-Garcia et al. [14] highlighted that manufacturers should be included in the loop, in order to create more resilient devices. The authors propose a certification methodology that delivers a measurable evaluation of IoT devices security, as well as an automatic security assessment. Moreover, they noted the lack of an IoT vulnerability database, which would enable better automatic security tests. Once these problems are amended, manufacturers could include in contracts useful information about compliance to security standards. As an example, a contract could include the last time the device software was verified against the vulnerability database, or the security level assigned by the automatic evaluation tool.

Kuusijärvi et al. [15] proposed to strengthen IoT security through a network edge device (NED), a secure device which stores the user-defined policies and enforces them on resource-constraint IoT devices. However, this kind of approach fails to identify specific requirements of the devices (i.e., they do not envision anything like a contract), offloading on the end-user the cumbersome task of defining fine-grained policies.

Other researchers proposed Fog-based policy enforcement approaches to solve different problems in the IoT world, for example, ensuring data privacy [16] and providing secure resource orchestration in Fog computing [17]. Similar to our work, they share the necessity of enforcing policies at the Fog layer over devices that might not be compliant by design.

Cisco manufacturer usage descriptions (MUD) [8] shares a number of common traits with our proposal. First and foremost, they envision a file that acts as a contract and states the device requirements. Second, the MUD file is parsed by a special node within the network (the MUD server) which defines appropriate access control lists (ACLs). However, a MUD file is pretty restrictive, as it barely describes basic information like allowed protocols and reachable hosts. Moreover, each MUD-compliant device does not carry the contract itself, but a simple URI that points the MUD manager to the online MUD file: Lack of Internet access would entail the total incapability of joining a network. Moreover, it is unclear how the contracts should be parsed, verified, and treated with respect to a security policy. Here, the key S × C concept of contract/ policy matching, as well as a way to enforce policies on untrustworthy devices, seems to be missing.

Other researchers built on MUD. Hamza et al. [18] tried to undertake the problem of enforcing policies by means of combination with a software defined network (SDN). In this context, the authors produced a translation from MUD policies to routing rules, aiming to implement the result into network switches. Again, they also noted that flow-based rules can be a first step to identify volumetric attacks, but they are not perfect. In particular, if such attacks happen on intended ports, MUD alone cannot be enough to identify the ongoing attack.

Another work proposed an automatic process, mainly (but not exclusively) targeted at manufacturers, to extract a MUD file from an IoT device traffic trace. Moreover, they sketched the necessity of formally matching MUD files with network security policies [11].

## 10. Future Work

As future work, we plan to further expand on the matching routines and the dynamic evolution of an S × C based environment. For instance, what happens when a new device receives a software update, or when contracts and policies receive updates, or when we try to remove from the network a device which is critical for other functionalities. Moreover, we plan to mitigate the threats discussed in Section 8. In particular, we will focus on reducing the susceptibility of the Fog node to disruptive attacks.

With this in mind, we are currently developing a Java prototype of an S × C IoT system. Our goal is to show the feasibility of our approach and demonstrate a few key points. First, we want to show that our system has the capability of detecting inconsistencies. Second, we want to show that our system solves or signals each and every inconsistency. As a consequence of the first two steps, we want to show that an S × C system can maintain consistency over time. Last, we want to show that such a system allows only intended communication flows and prevents unintended data leaks.

**Figure 7:**
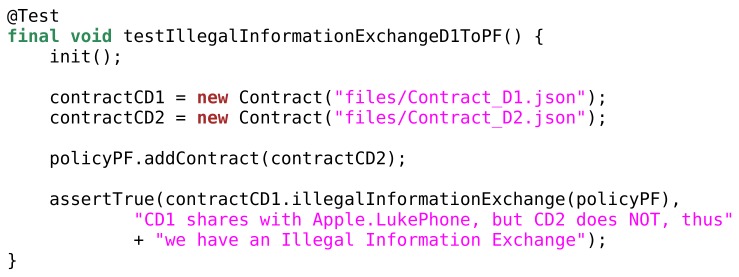
Code snippet that tests the implementation of IllegalInformationExchange.

As an example of the ongoing implementation, in Figure 7 we show a Java code-snippet. In particular, this function tests whether our implementation of IllegalInformationExchange is correct.

Last but not least, we are working on extending our framework for integrating devices that are not S × C compliant (that is, devices that do not have a contract). We believe this represents a fundamental point for the adoption of our approach.

## 11. Conclusions

In this paper, we have proposed a novel approach that combines security-by-contract (S × C) and Fog computing to tackle the issue of IoT insufficient security configurability.

In our proposal devices are equipped with security contracts that can be verified against the security policy stored within the Fog node. In turn, this allows for tractable contracts and policies, efficient matching routines, and trustworthy IoT environments. By means of a running case scenario and a number of illustrative examples, we have defined all the necessary pillars to develop our proposal, from basic security rules up to consistent security policies. Moreover, we have provided pseudo-code algorithms which show the feasibility of our approach.

## Figures and Tables

**Table 1:** Roles, actions, and implications in the S × C setting.

	Actions	Implications
User	Connects his S × C device to the network	Completely transparent for the user
Administrator	Manages the S × C network policy	Small overhead, in the form of policy creation and management
Manufacturer	Write device contract and provides PoC	Overhead in the form of contract and PoC certificate creation

**Table 2:** Security rule structure.

Device D	The device name d and manufacturer M of the device, expressed as M.d.
Domain Dom	The domain where the rule applies. For instance, Dom = LAN for rules that apply within the local network, or Dom = * for rules that apply to any domain.
Shares	List of devices that the device D can interact with, in the domain Dom . We use * to denote that anything applies. Examples: M.* specifies any device from a specific manufacturer M . Similarly, *.* (or simply *) specifies any device from any manufacturer.
Provides	List of services s${}_{1}$, …, s${}_{n}$, n≥0, that the IoT device D provides to the devices in Shares$\left(D_{}\right)$.
Requires	List of services s${}_{1}$, …, s${}_{m}$, m≥0 that the IoT device D requires to function.

**Table 3:** Security rule R${}_{A1}$.

	Rule R${}_{A1}$
D	D-Link.933L
Dom	*
Shares	Apple.LukePhone
Provides	SetDayNight
Requires	-

**Table 4:** Security rule R${}_{A2}$.

	Rule R${}_{A2}$
D	D-Link.933L
Dom	*
Shares	-
Provides	-
Requires	Philips.HueWhite.On

**Table 5:** Security rule R${}_{B1}$.

	Rule R${}_{B1}$
D	Philips.HueWhite
Dom	LAN
Shares	Philips .*
Provides	On *,* Bri *,* Hue
Requires	Philips.HueMotion.Presence

**Table 6:** Security rule R${}_{\mathrm{MA}}$.

	Rule R${}_{\mathrm{MA}}$
D	D-Link.933L
Dom	*
Shares	-
Provides	SetDayNight
Requires	-

**Table 7:** Security contract C${}_{A}$.

	Rule R${}_{A1}$	Rule R${}_{A3}$
D	D-Link.933L	D-Link.933L
Dom	*	LAN
Shares	Apple.LukePhone	Apple.LukePhone
Provides	SetDayNight	SetDayNight*,* AdminPanel
Requires	-	-

**Table 8:** Security contract C${}_{B}$.

	Rule R${}_{B1}$	Rule R${}_{B2}$
D	Philips.HueWhite	Philips.HueWhite
Dom	LAN	LAN
Shares	Philips .*	*.*
Provides	On *,* Bri *,* Hue	On *,* Bri
Requires	Philips.HueMotion.Presence	-

**Table 9:** Security contract C${}_{\mathrm{IB}}$.

	Rule R${}_{B3}$	Rule R${}_{B2}$
D	Philips.HueWhite	Philips.HueWhite
Dom	LAN	LAN
Shares	Philips .*	*.*
Provides	On	On *,* Bri
Requires	Philips.HueMotion.Presence	-

**Table 10:** Security policy P${}_{A}$.

	Rule R${}_{A1}$	Rule R${}_{B1}$	Rule R${}_{B2}$
D	D-Link.933L	Philips.HueWhite	Philips.HueWhite
Dom	*	LAN	LAN
Shares	Apple.LukePhone	Philips .*	*.*
Provides	SetDayNight	On, Bri, Hue	On *,* Bri
Requires	-	Philips.HueMotion.Presence	-

**Table 11:** Security policy P${}_{B}$.

	Rule R${}_{A1}$	Rule R${}_{B2}$	Rule R${}_{\mathrm{Admin}1}$
D	D-Link.933L	Philips.HueWhite	Philips .*
Dom	*	LAN	Internet
Shares	Apple.LukePhone	*.*	-
Provides	SetDayNight	On, Bri	-
Requires	-	-	D-Link.933L.SetDayNight

**Table 12:** Illegal information exchange C${}_{D_{1}}$ *⇏* P${}_{F_{}}$.

	C${}_{D_{1}}$	C${}_{D_{2}}$ ∈ P${}_{F}$
D	D-Link.933L	Philips.HueMotion
Dom	LAN	LAN
Shares	Apple.LukePhone	D-Link.933L, Philips.HueWhite
Provides	SetDayNight	On, Bri
Requires	Philips.HueMotion.On	-

**Table 13:** Inconsistent security policy P${}_{\mathrm{IB}}$.

	Rule R${}_{A1}$	Rule R${}_{B2}$	Rule R${}_{\mathrm{Admin}2}$
D	D-Link.933L	Philips.HueWhite	Philips.HueWhite
Dom	*	LAN	LAN
Shares	Apple.LukePhone	*.*	*.*
Provides	SetDayNight	On, Bri	On *,* Bri *,* Hue
Requires	-	-	-

**Table 14:** Security contract C${}_{\mathrm{Echo}1}$.

	Rule R${}_{E1}$
D	Amazon.Echo
Dom	LAN
Shares	*.*
Provides	VocalInput
Requires	-

**Table 15:** Security contract C${}_{\mathrm{Echo}2}$.

	Rule R${}_{E2}$
D	Amazon.Echo
Dom	*
Shares	*.*
Provides	VocalInput
Requires	-

**Table 16:** Inconsistent security policy P${}_{\mathrm{FR}}$.

	Rule R${}_{\mathrm{FR}1}$	Rule R${}_{E2}$
D	Amazon.Echo	Amazon.Echo
Dom	*	*
Shares	*.*	*.*
Provides	-	VocalInput
Requires	-	-

**Table 17:** Inconsistent security policy P${}_{\mathrm{OORT}}$.

	Rule R${}_{\mathrm{Plug}}$	Rule R${}_{\mathrm{Hub}}$	Rule R${}_{\mathrm{Sensor}}$
D	OORT.Plug	Samsung.Hub	Samsung.Sensor
Dom	LAN	LAN	LAN
Shares	*.*	OORT.Plug	Samsung.Hub
Provides	*	OnOff	OpenClose
Requires	Samsung.Hub.OnOff	Samsung.Sensor.OpenClose	-

**Table 18:** Security contract C${}_{\mathrm{OORT}\mathrm{Lock}}$.

	Rule R${}_{L1}$
D	OORT.Lock
Dom	*
Shares	*.*
Provides	OpenClose
Requires	-

**Table 19:** Possible attacks on IoT devices (with vs without S × C).

Action	Without S × C	With S × C
Compromised contract; Unaltered code; Unaltered PoC	Do not apply, no contract exists -	Fog node rejects device at installation time ✓ Fog node discovers the breach at runtime as soon as the device behaves unexpectedly ✓
Compromised PoC; Unaltered code; Unaltered contract	Do not apply, no PoC exists -	Fog node rejects device at installation time ✓
Compromised code; Unaltered contract; Unaltered PoC	Fog node has no way to detect unintended behaviours. Device is erroneously accepted ✗	Fog node detects at runtime the breach as soon as the device behaves in a way that violates the policy ✓
Behaviour violating policy; Matching code, contract, and PoC	Fog node has no way to detect unintended behaviours. Device is erroneously accepted ✗	Fog node detects at runtime that the behaviour does not match the network policy. Device is rejected ✓
Behaviour complying with policy; Matching code, contract, and PoC	Fog node has no way to detect unintended behaviours. Device is erroneously accepted ✗	Fog node cannot detect at runtime that the behaviour does not match the network policy. Device is erroneously accepted ✗

**Table 20:** Consistent security policy P${}_{\mathrm{OORTCons}}$.

	Rule R${}_{\mathrm{Plug}}$	Rule R${}_{\mathrm{Hub}}$	Rule R${}_{\mathrm{Sensor}}$
D	OORT.Plug	Samsung.Hub	Samsung.Sensor
Dom	LAN	LAN	LAN
Shares	*.*	OORT.Plug	Samsung.Hub, OORT.Plug
Provides	*	OnOff	OpenClose
Requires	Samsung.Hub.OnOff	Samsung.Sensor.OpenClose	-

**Table 21:** Possible attacks on fog node and communication channels (with vs without S × C).

Action	Without S × C	With S × C
Compromising policy	Do not apply, Fog node is not in charge of the security policy -	Compromised policy, but devices’ contracts and codes are unaltered. Attacker can only impede communications through a restricting policy. A more permissive policy cannot cause unexpected information leaks, devices still respect their own contracts ~
DoS attack on Fog node	Do not apply, Fog node is not in charge of the security policy -	Compromised system, but devices’ contracts and codes are unaltered. Devices can potentially negotiate M2M and keep working according to their contracts ~
Man-in-the-Middle (MitM) attack	Attacker can steal and alter data ✗	Attacker can steal and alter data ✗
